# Melamine aggravates neurotoxicity in D‐galactose‐induced aging SH‐SY5Y cells, elevating Alzheimer's risk

**DOI:** 10.1002/alz70855_102250

**Published:** 2025-12-23

**Authors:** Juhi Goyal, Nitish Rai

**Affiliations:** ^1^ Mohanlal Sukhadia University, Udaipur, Rajasthan, India; ^2^ Department of Zoology, University of Lucknow, Lucknow, Lucknow, India

## Abstract

**Background:**

Several findings have highlighted the importance of diet and ecotoxic compounds on aging. Melamine (Mel), a widely documented food adulterant, has demonstrated toxicity in multiple organs of the human body, including the brain. However, its neurotoxic effects on aging neurons remain unexplored. This study aims to bridge this gap by investigating the *in‐vitro* neurotoxic impact of Mel in a D‐galactose (DG)‐induced aging model of neuronal SH‐SY5Y cells.

**Method:**

In the present study, the SH‐SY5Y cells were administered a toxic dose of Mel and DG individually and in combination (Mel + DG) to determine their potential for neurotoxicity. The analysis involved measuring cell viability through MTT assay and morphological examination via neurite length assessment. Furthermore, we evaluated the antioxidant status of the cells by examining catalase (CAT), superoxide dismutase (SOD), and total antioxidant activities. Subsequent investigations of reactive oxygen species (ROS), mitochondrial membrane potential (MMP), and caspase‐3 (Casp3) activity were also performed.

**Result:**

The co‐administration with Mel and DG resulted in the highest cell death compared to individual treatments of Mel or DG and control untreated cells. The combined exposure of Mel and DG also led to significant neurite shrinkage and ROS accumulation, indicating exacerbated toxicity. Moreover, SOD, CAT, and total antioxidant levels were significantly reduced in the co‐treatment (Mel + DG) group than in Mel‐only or DG‐only treated group, showing excessively hampered antioxidant state. Additionally, the Casp3 activity was markedly elevated in the cell group jointly treated with Mel and DG, as compared to Mel or DG alone treated groups, signifying a heightened apoptotic reaction.

**Conclusion:**

This study provides early insights into the increased neurotoxic potential of Melamine (Mel) in an aging model of neuronal cells. The outcomes reveal that Mel consumption in the elderly may contribute to a higher risk of neurodegenerative disorders, such as Alzheimer's and Parkinson's diseases.

